# On‐Scanner Correction of Gradient Nonlinearity Bias for Accurate Assessment of Diffusion Heterogeneity Across Bone Sites in Myelofibrosis Patients

**DOI:** 10.1002/mrm.70273

**Published:** 2026-01-29

**Authors:** Dariya Malyarenko, Brian D. Ross, Johannes M. Peeters, Ajit Devaraj, Ramin Jafari, Humera Tariq, Kristen M. Pettit, Moshe Talpaz, Gary D. Luker, Thomas L. Chenevert

**Affiliations:** ^1^ Radiology University of Michigan Ann Arbor Michigan USA; ^2^ MR Clinical Science Philips Healthcare Best the Netherlands; ^3^ MR Clinical Science Philips Cambridge Massachusetts USA; ^4^ Internal Medicine University of Michigan Ann Arbor Michigan USA

**Keywords:** ADC nonuniformity, bone marrow, gradient nonlinearity, myelofibrosis, nonuniform diffusion weighting

## Abstract

**Purpose:**

To improve accuracy of apparent diffusion coefficient (ADC) measurement across different bone‐marrow (BM) sites for myelofibrosis (MF) patients.

**Methods:**

Vendor‐provided ADC gradient nonlinearity correction (GNC) was implemented for 41 MF study subjects on a 3T clinical scanner. The degree of bias correction was assessed on b‐maps across the BM regions‐of‐interest defined for femoral trochanter, posterior ilium and spine vertebrae of all subjects. Bias variability from subject repositioning was evaluated for five subjects with longitudinal scans using Bland–Altman analysis. BM ADC with and without GNC were compared in 22 subjects with MF grade > 0 (iliac fat fraction < 40%). The GNC effect on ADC heterogeneity trends across bone sites was assessed using paired t‐test and correlation to MF‐grade.

**Results:**

The observed bias was substantial across BM sites ranging from −10% for edge vertebrae to +8.4% for trochanter across all subjects with a 13.7% intra‐subject median range. The effect of irreproducible positioning in longitudinal scans was ±4.2% bias (95% limits‐of‐agreement) with range of ±8.5% (highest for L1 and L5). By removing bias, GNC improved ADC accuracy and longitudinal reproducibility and emphasized biological heterogeneity for femoral trochanter versus ilium and edge vertebrae (T11‐L1, L5, and S1). GNC revealed significant ADC differences between trochanters and edge vertebrae (*p* = 0.007), while edge vertebrae and ilium ADC became more uniform (*p* = 0.07), with heterogeneity and values correlated to MF‐grade.

**Conclusion:**

On‐scanner correction of gradient nonlinearity bias in bone marrow ADC reduces technical nonuniformity and variability and allows accurate and reproducible characterization of heterogeneous disease for myelofibrosis patients.

## 1 Introduction

An ongoing single‐site clinical trial is investigating quantitative MRI for longitudinal monitoring of myelofibrosis (MF) [1] progression and treatment efficacy. Comprehensive MRI examination of the bone marrow (BM) space (primary MF disease site) provides a desirable non‐invasive alternative to posterior ilium BM biopsies, used in current standard of care [2, 3] that are painful and prone to sampling errors [4, 5]. To circumvent challenges of BM analysis in patients, oncologists largely rely on indirect measures of disease involvement including spleen volume and blood analysis [3, 6]. In MF patients with advanced disease, diagnostic value of needle biopsies of bone marrow is often limited by recovering sparse or no tissue [4, 5] leading to inconsistent perception for semi‐quantitative MF descriptors, such as cellularity and fibrosis grade [7]. Even successful biopsies sample only a small fraction of body BM (from iliac crest), insufficient to assess spatial disease heterogeneity [8, 9].

The apparent diffusion coefficient (ADC) metric derived from diffusion weighted imaging (DWI) reflects changes in tissue cellularity [10, 11] potentially associated with disease progression and treatment response [12, 13, 14, 15]. Hypercellular bone marrow in myeloproliferative neoplasm (MPN) and associated fibrosis in MF [2, 3] can paradoxically increase ADC values [9, 10, 11] in comparison with relatively low ADC observed in healthy bone marrow [16, 17, 18] due to its naturally large fat fraction and trabecular bone environment. As a quantitative probe of BM cellularity and fibrosis, the ADC metric is being evaluated as a potential alternative to biopsy for quantitative assessment of MF disease extent, heterogeneity and therapy response [9, 13, 14, 15]. Accurate ADC measurements hold potential to improve diagnostic yield of MF MRI examinations with increased anatomic coverage and patient comfort compared to needle biopsy performed in iliac crest. To accurately assess biological disease heterogeneity across bone marrow sites using ADC, technical biases that cause spatially varying ADC values need to be corrected.

Spatially dependent bias in diffusion weighting due to systematic gradient nonlinearity (GNL) results in incremental modulation of ADC maps over the three‐dimensional imaged volume [19, 20]. The relatively large field of view (FOV˜400 × 400 mm^2^) required to encompass the lumbar spine and pelvic bones for MF imaging leads to substantial non‐uniformity of diffusion weighting (DW) *b*‐values induced by GNL [19, 20, 21]. Deviation from nominal *b*‐value due to GNL bias depends on anatomy location and increases with distance from magnet isocenter [21, 22, 23]. Thus, uncorrected GNL bias confounds analysis of biological MF disease heterogeneity based on BM ADC measurement. Furthermore, potential variability in patient positioning over longitudinal studies can obscure true ADC change due to biological alteration preventing confident evaluation of therapy response effects [24, 25]. Correction of systematic GNL bias for BM ADC [21] helps eliminate errors due to shifts of bone anatomy between imaging points for individual patients to tighten confidence intervals [25] for detection of significant biological change.

Improved ADC accuracy after retrospective (offline) correction for system‐specific GNL bias [19, 20] has been demonstrated for off‐center anatomy in previous studies [22, 26]. Through an ongoing academic industrial partnership project with MRI vendors, the developed technology is being implemented on clinical systems. Recently, the FDA‐approved option for GNL correction (GNC) of ADC was provided on clinical scanners allowing prospective (online) application during patient scans [21, 23]. Several clinical studies have demonstrated promising effect of the correction for ADC measurements for bone metastasis [21] and head‐and‐neck tumors [27]. These emerging online GNC implementations are preferred over retrospective correction due to simplified logistics for multi‐system and multiple time point imaging [22, 26] but require validation for practical integration with a clinical workflow.

Our previous MF imaging study observed high heterogeneity of ADC parameters across the lower spine and pelvic bone marrow space of MF patients with significant correlation of ilium ADC to fibrosis grade [9]. To further disentangle biological effects from technical biases at different bone sites and enhance longitudinal reproducibility for future therapy response evaluation at multiple imaging points based on bone‐marrow ADC measures, this work extends application of on‐scanner GNC in a prospective myelofibrosis clinical imaging trial. Here we evaluate the GNC performance to reduce technical variability and artefactual spatial nonuniformity of ADC measurements in the bone‐marrow of myelofibrosis patients to enable accurate and reproducible assessment of biological disease heterogeneity and therapeutic effects across bone sites.

## 2 Methods

### 2.1 MF Subject DWI

**FIGURE 1:**
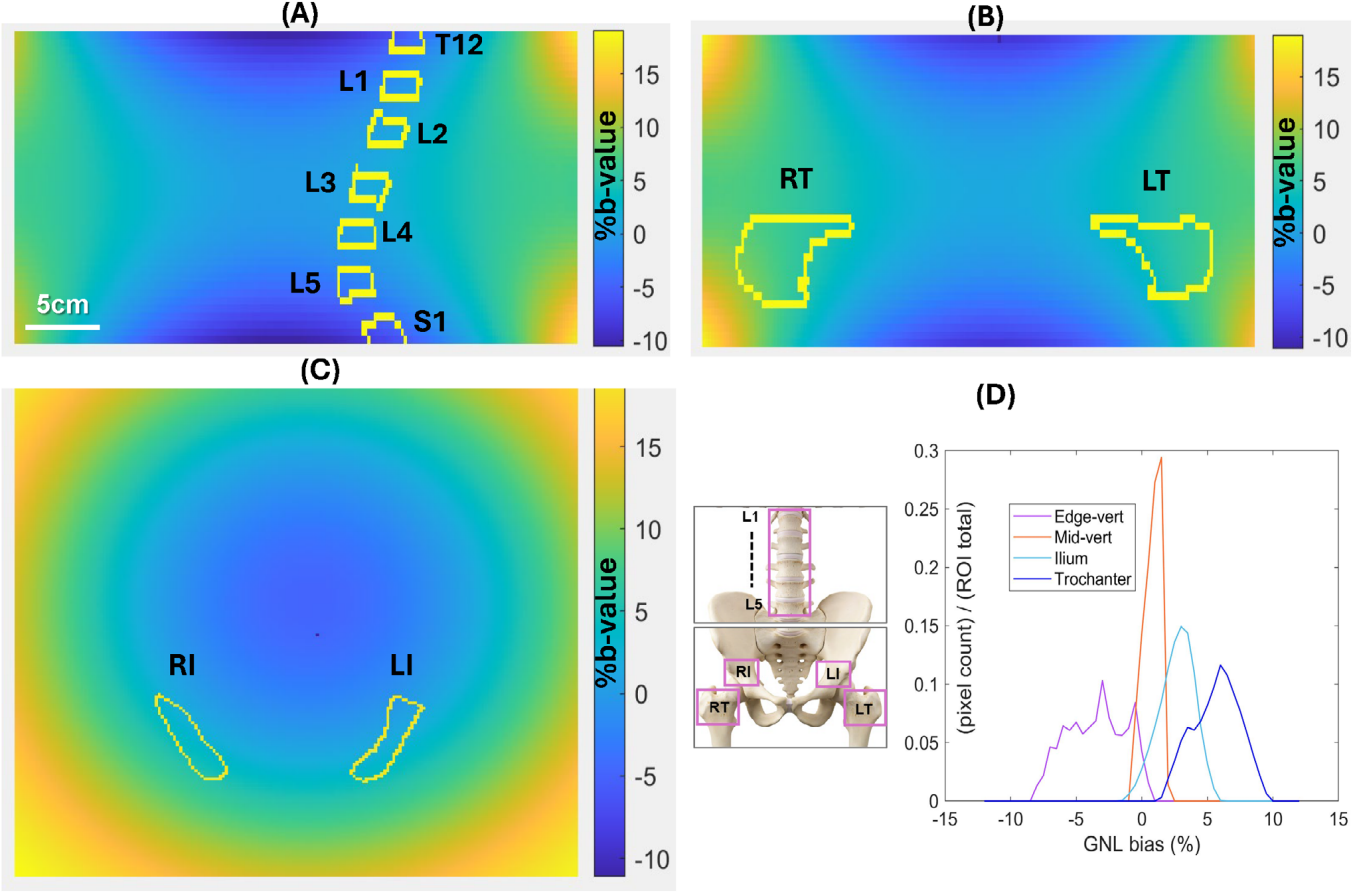
Percent GNL bias maps (for b‐values) with region‐of‐interest (ROI) overlay for a representative myelofibrosis subject are shown in three orthogonal image planes (A: Sagittal, B. coronal; C: Axial.) The color‐bars indicate the GNL bias scale (%) with respect to nominal b‐value at isocenter (zero bias). Middle insert shows imaged bone sites for two stations (top: Lumber spine and bottom: Pelvic‐proximal femur) with pink boxes identifying bone sites labeled in subsequent figures as right/left trochanter: RT/LT, right/left ilium: RI/LI, and L‐spine vertebrae: L1‐L5. The bias histograms (bin size 0.5%, normalized to total ROI volume) for this subject ROIs are shown across bone sites in (D) for combined mid‐vertebrae (L2‐L4), edge‐vertebrae (T12, L1, L5, S1) and right–left ilium and trochanter (color‐coded in the legend).

For the NCT01973881 trial [1, 9], MRI examinations were performed for IRB‐consented study subjects on a single 3T MRI scanner (Ingenia, Philips, Best, Netherlands). Fat suppressed trace‐DWI were acquired using standardized single‐shot EPI sequences with *b* = 0, 800 s/mm^2^ in two imaging stations with 12–23 cm table offsets to cover lumber spine and ilium to proximal femur bones (Figure 1, middle insert). Details on acquisition parameters are summarized in Table S1. For this study, 41 subjects were scanned between 2021 and 2025 with delayed reconstruction of ADC maps with and without vendor‐provided GNC implemented on the scanner according to previously developed method [20] based on GNL tensor model (summarized in the Figure S1). The default image noise filter was disabled prior to delayed reconstructions to facilitate isolation of GNC effects. Over the course of the GNC study, five subjects had longitudinal scans approximately 3–6 months apart (2 timepoints for two subjects, and 4, 5, and 6 timepoints for remaining three subjects, respectively). Thirty‐three test–retest scan pairs were formed for GNL bias maps from these longitudinal scans. Quantitative proton‐density fat‐fraction (PDFF) mapping was also performed as a part of MRI exam [9, 28] using vendor‐provided 3D mDIXON‐QUANT protocol with six‐echoes (Table S1). Twenty‐two subjects with ilium fat‐fraction < 40% were selected for ADC GNC analysis (as detailed below). Patient demographics for the study are summarized in Table S2. The ADC, ADC_GNC_ and b‐maps were reconstructed on the scanner and converted from DICOM to meta‐image header (MHD) ITK‐compatible format with appropriate “display value” scaling [29]. The vendor‐provided b‐map DICOMs were dimensionless (normalized to nominal *b*‐value) and scaled to 1E4 at the isocenter, while ADC was in μm^2^/ms units.

### 2.2 B‐Map Validation

The b‐maps and on‐scanner ADC_GNC_ were pre‐validated using a coronal DWI of torso‐size sodium polyacrylate aqueous phantom (Figure S1) [30]. Fractional ADC bias‐percent maps were generated by normalized subtraction before and after GNC: 100%* (*ADC* − *ADC*_*GNC*_)/*ADC*_*GNC*_ and compared to b‐maps for coronal phantom slice to ensure less than 1% deviation from the system model within typical FOV for MF subjects (encompassing pelvic and lower spine bone sites). Vendor‐generated b‐maps were previously verified using system spherical harmonics (SPH) information and a GNL tensor model described elsewhere [31], according to published GNC formalism [20] summarized in Figure S1. Subject‐specific vendor‐generated 3D b‐maps were converted to predicted GNL bias maps defined as 100% × ((b‐map/1E4) − 1) scaled such that the bias is zero at magnet isocenter and saved in MHD format. Bias maps were identical for lower spine and pelvic imaging stations (Figure 1, insert), confirming that scan table offset was properly considered in GNC and b‐map generation. Note, while on‐scanner bias maps were created for each subject's scan, they are based on fixed system GNL tensor derived from SPH coefficients [20, 31, 32] thus are effectively noiseless and represent corrected GNL nonuniformity bias in nominal *b*‐values (Figure S1).

### 2.3 GNL Bias Analysis

The bone marrow ROI labels (e.g., Figure 1) were defined for bi‐lateral ilium and trochanter, and for lower spine vertebral bodies as described in Supporting Information and applied in 3D Slicer to b‐maps of 41 subjects to analyze GNL errors as a function of a bone site and patient positioning (Figure 1A–C). The GNL bias statistics (volume, mean, median, range) for bone marrow ROIs were generated in 3D Slicer for each subject and bone site. The mean corrected bias was estimated for all subjects and time points across the spine vertebral bodies (lumber L1‐L5), right and left ilium (RI, LI) and femoral‐trochanter bone (RT, LT) locations (Figure 1A–C). The bias measurement was performed for all subjects in right and left ilium (RI & LI) and lumbar L1‐L4 vertebrae. Two study subjects had hip implants (one bilateral, and one in the left hip), preventing corresponding trochanter ROI definitions that resulted in 40 RT and 39 LT measurements. The imaged vertebra numbers varied due to different patient height and S‐I positioning. Forty subjects had L5, 30 also had thoracic T12, seven—sacrum S1, and five—thoracic T11 vertebrae in the DWI of the upper station scan.

The bias‐induced nonuniformity was quantified as the difference between maximum and minimum bias across bone sites of the same subject. The repeatability analysis was performed for longitudinal b‐maps only since bone size and system GNL maps are known to be constant over the term of this experiment [32]. Therefore, “repeatability” in this context reported on how reproducibly a given subject is positioned and landmarked over longitudinal scan sessions months apart by experienced research MRI technologists (*N* = 2). For GNL bias repeatability analysis, 33 test–retest pairs were analyzed for RI, LI, RT, LT and L1‐L5, and 17 pairs for T12 bone locations. The Bland–Altman limits‐of‐agreement (LOA) were assessed as 1.96*SD(difference). Note, ADC repeatability could not be estimated from these longitudinal scans due to potential true biological changes in BM ADC occurring during treatment and/or disease progression.

### 2.4 ADC GNC Analysis

ADC versus ADC_GNC_ was analyzed for the 1st time point scan of subset of 22 subjects with ilium fat‐fraction < 40% (median [range] = 19 [4, 38]%). This subset was selected to ensure robust ADC measurement for MF subjects with sufficient signal‐to‐noise ratio (SNR) and contrast‐to‐noise ratio (CNR). For the subjects with > 40% ilium fat, the mode ADC was < 0.3μm^2^/ms and on the order of standard deviation (SD) such that ADC histograms appeared substantially truncated (Figure S2), preventing reliable statistical analysis of GNC effect. Among remaining 19 study subjects (including two healthy controls), ilium fat fraction was median [range] = 53 [46, 73]%. The mean ADC values for healthy controls were 0.35 ± 0.23 μm^2^/ms (Figure S2) consistent with prior studies [16, 17, 18]. There was no significant difference in age of the subgroups confirmed by one‐way analysis of variance (*p* = 0.52). The female to male ratio was about 2:1. Except for one subject with unknown grade and one with MF grade zero, the group selected for ADC analysis had MF grade > 0 confirmed by biopsy (Table S2).

The ADC GNC performance was compared to the combined bone marrow ROI histograms of middle vertebrae (MV: L2 & L3 & L4) versus edge vertebrae (EV: T12 & L1 & L5 & S1), as well as, combined right and left ilium (I: RI & LI), versus right & left trochanter (T: RT & LT). The ADC ROI histogram analysis included ADC > 0.01 μm^2^/ms to leave out voxels with limited contrast‐to‐noise. Histogram mean, median and SD values before and after GNC were tabulated for statistical analysis. The potential confounding effect of unsuppressed fat on ADC was evaluated from mean ADC correlation to PDFF for ilium and femur bone sites across 22 selected subjects. The relations between ADC at different bone sites and BM ROI volume, age, gender and MF grade across patients were assessed using Pearson correlation coefficient, R. (The small number of subjects per MF grade (6–7, Table S2) did not warrant comparisons for individual grade sub‐groups.) The boxplots were used for visualization. Paired two‐sided *t*‐test was used for GNC effect assessment and single‐sided *t*‐test for ADC heterogeneity comparisons between bone sites across studied MF subjects. The significance level was set to *p* < 0.05 for correlation and single‐parameter comparisons, and Bonferroni corrected *p* < 0.008 was used for multiple comparisons across bone sites. The average ADC heterogeneity across bone sites was quantified by coefficient of variance (CV). The deviations from average heterogeneity trend at individual bone sites were explored in relation to clinical subject characteristics. All ADC image processing, visualization and statistical analysis were performed in Matlab R2019b (Mathworks, Natick, MA).

## 3 Results

Figure 1A–C shows an example of GNL bias maps with bone‐site ROI overlays for a representative MF study subject in three imaging planes. Similar to Figure S1 for phantom, the spatial b‐map bias patterns scale positively along right–left (R‐L) and anterior–posterior (A‐P) directions, and negatively for superior–inferior (S‐I) anatomy offsets from magnet isocenter (zero %b‐value bias). The bias pattern asymmetries within the FOV reflected R‐L shifts in patient positioning for axial and coronal views (Figure 1B,C) and A‐P table elevation for sagittal view (Figure 1A). The sample bias histograms in Figure 1D illustrate typical GNL effect on applied b‐value across the imaged bone sites consistent with system GNL model for the specific ROI positions. As predicted by known system GNL spatial nonuniformity patterns (Figure S1C), *b*‐value bias should be predominantly positive for ilium and trochanter sites, and negative for edge vertebrae (T12, L1, L5, and S1) relative to negligible bias in central L‐spine (L2‐L4 mid‐vertebrae). The observed *b*‐value bias is largely negative (range: −8% to 1%) for edge vertebrae (EV) and nominally negligible (−1% to +2%) for mid‐vertebrae (MV), going slightly positive (−1% to +6%) for ilium (I) and higher (+1% to 10%) for the trochanter (T). The broader histogram width observed for trochanter and EV bias apparently reflects wider distribution of voxel positions within the corresponding ROIs.

**FIGURE 2:**
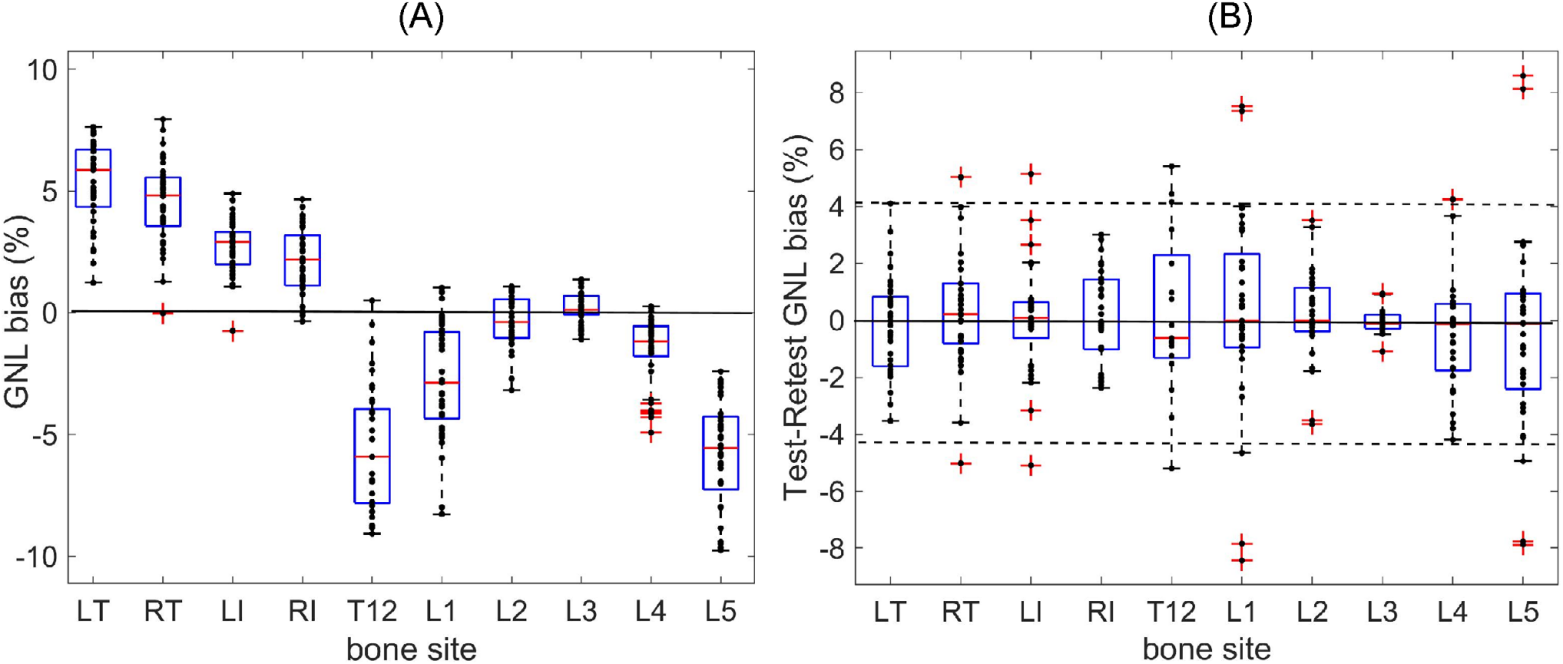
(A) Box‐plot summary of ROI‐average GNL bias corrected for baseline scans of 41 myelofibrosis patients at different bone sites labeled on horizontal axis for right/left trochanter (RT/LT), ilium (RI/LI), L‐spine vertebrae (L1‐L5) and thoracic vertebrae (T12). Subject‐specific values are depicted by dots along the middle vertical line. Box boundaries correspond to the 1st and 3rd data quartiles (Q1 and Q3) and mid‐lines show median values. Pluses indicate outliers beyond 99% confidence intervals (error‐bars). (B) Right panel shows b‐map test–retest results for five subjects with longitudinal scans. The dashed lines correspond to limits‐of‐agreement (±4.2%) across all bone sites with respect to 0.01% average bias marked by a solid line.

Figure 2 summarizes GNL bias corrected at individual bone sites across study subjects and time points. The overall corrected bias ranged from −10% (L5) to +8.4% (RT) (median of −6.5% at T12 to 6% at LT) across patients (Figure 2A) and from 8.9% to 16.5% (median of 13.7%) between trochanter and edge vertebrae of the same patient. The lowest median corrected bias (0.02%) was evident for L3 and followed negative parabolic pattern for T12‐L1 and L4‐L5 (consistent with phantom S‐I pattern, Figure S1C). For seven subjects that had sacrum S1 and for 5 that had thoracic T11 vertebrae in their single time‐point DWI, corrected biases were similar to those observed for L5 and T12: median [range] for S1 was −7% [−6%, −8.6%], and for T11 was −6.5% [−4.4%, −8.6%] (boxplots not included in Figure 2). Notably, the apparent difference between corrected bias in LT and RT was significant (*p* < 0.001), suggesting prevalence of left shifts among patients likely associated with the loading table side. In contrast, the LI, RI bias differences were not significant (*p* > 0.2) since these bone sites are closer to midline.

The larger bias spread (box‐width) was observed for T12, L1, L5, LT and RI (Figure 2A), consistent with effect of patient sizes. Bias spread prevalence at the same bone sites is observed for test–retest analysis in Figure 2B. For test–retest scans T12 had 17 measurements (out of 33), while the S1 and T11 were not repeatedly measured, suggesting at least a single vertebra shift common for patient repositioning along SI. The observed test–retest bias variability range (from −8.4% to +8.6%) is the highest for L1 and L5 (with median of −0.08% across all time points). The best repeatability (within ±1%) is observed for L3. The Bland–Altman plots in Figure S3 likewise illustrate wider limits of agreement for combined (L1, L5) edge‐vertebrae (±6.9%, Figure S3D), bi‐lateral trochanter (±4.1%, Figure S3A) and ilium (±3.7%, Figure S3B) compared to (L2, L3) mid‐vertebrae (±3%, Figure S3C). Across all bone sites (Figure 2B), the test‐rests bias is negligible (0.01%), however, rather broad limits of agreement (±4.2%) suggest that uncorrected GNL bias could cause substantial ADC variability for longitudinal subject scans.

**FIGURE 3:**
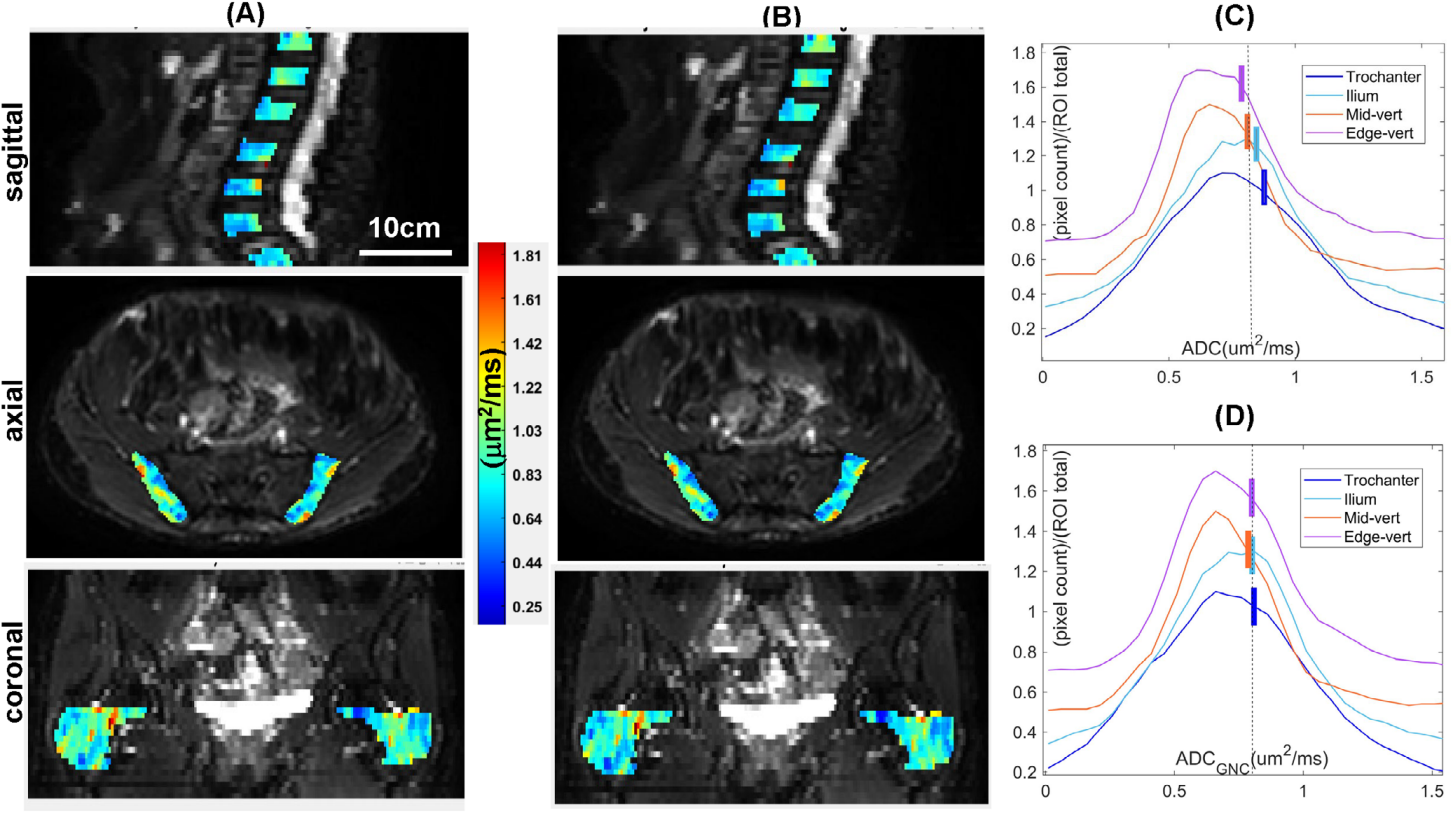
The ADC map ROI overlays on *b* = 0 s/mm^2^ DWI are shown for a representative myelofibrosis study subject in three orthogonal planes (arranged vertically) before (A) and after (B) gradient nonlinearity correction (GNC). The ADC maps were smoothed with 3‐point median filter. Color‐bar indicates the common ADC scale. The corresponding ADC histograms (bin size 0.05 μm^2^/ms, normalized to total count) are shown before (C) and after GNC (D) as stack plots (vertically offset for clarity) across trochanter, ilium, mid‐vertebrae (L2‐L4), and edge‐vertebrae (T12, L1, L5, and S1) bone sites (color‐coded in the legends). The colored notches mark the mean ADC values with respect to nominally bias‐free ADC(L3)‐reference (dashed vertical line).

Figure 3A,B illustrate GNL‐induced nonuniformity of *b*‐value introducing false heterogeneity convolved with biologic heterogeneity in bone marrow ADC and substantial ADC errors for off‐center bone anatomy (e.g., trochanter and edge‐vertebrae, Figure S4A inserts). The median ADC values for ROI histograms (Figure 3C,D) for combined bi‐lateral ilium and trochanter, as well as, edge and mid‐vertebrae, were slightly lower than mean values (notches) reflecting the presence of skew toward higher ADC values. The effect of GNC for this study subject (Figures 3B,D and S4A) manifests as improved ADC uniformity between edge vertebrae (0.78 ± 0.01 μm^2^/ms) and femoral trochanter (0.87 ± 0.01 μm^2^/ms) by 11% ± 1% (0.81–0.82μm^2^/ms after GNC). The correction of positive GNL bias in trochanter is contrasted with negative GNL bias in T12 in the Figure S4A inserts, leading to more uniform ADC after GNC across bone sites.

As expected, the average degree of ADC heterogeneity across bone sites for this subject was reduced by GNC (from CV = 4% to CV = 1%). Similar low ADC heterogeneity across bone‐sites (CV < 9%) after GNC was observed in 4 more subjects (predominantly females with MF grade 2). Half of MF subjects (13/22) exhibited intermediate ADC heterogeneity of 10% to 20%, while highest heterogeneity (CV = 23% to 30%) was observed for 4/22 subjects (male, MF grade 3), with two having their CV increased by 2% after GNC. Overall, no significant correlation was observed between CV and age (*p* = 0.13) or MF grade (*p* = 0.09), while female MF subjects showed significantly lower (*p* = 0.0044) average ADC heterogeneity across bone sites (CV = 10% ± 5%) versus male subjects (CV = 20% ± 8%).

**FIGURE 4:**
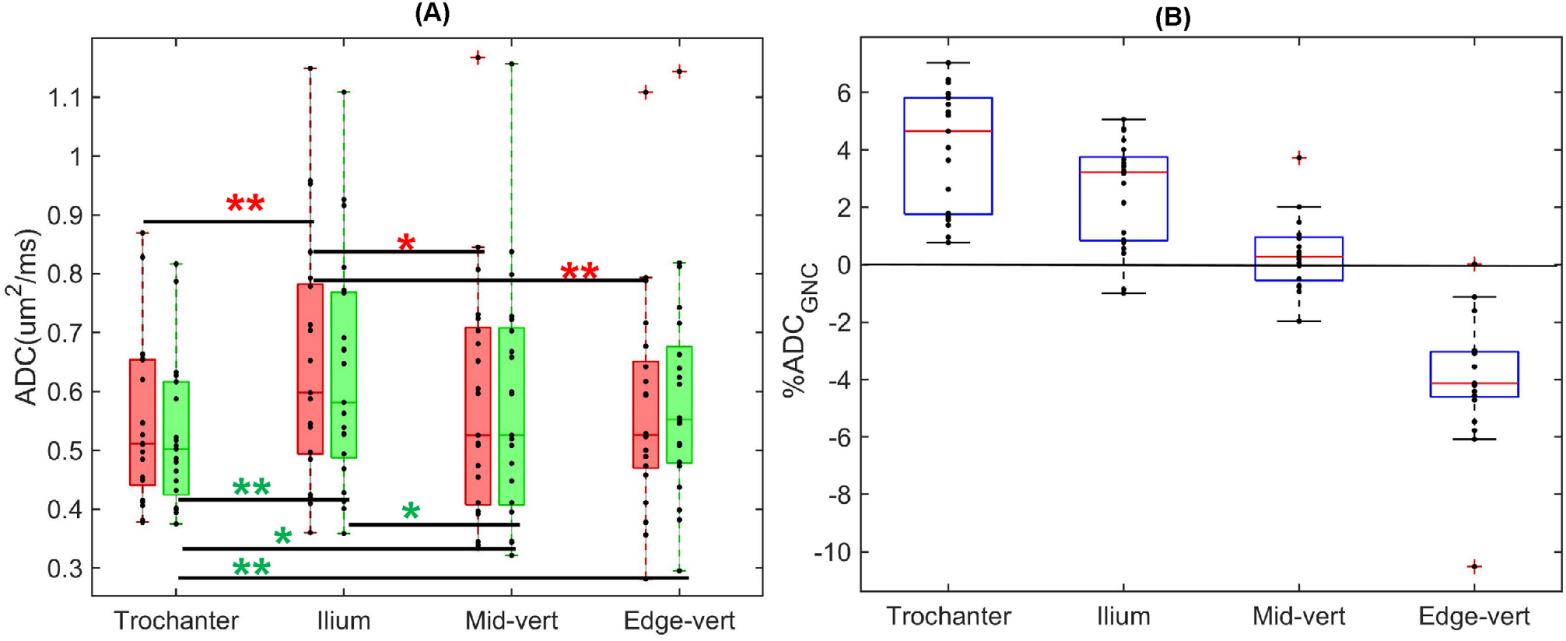
(A) Box‐plot summary of GNC effect on mean ADC (red—before correction, green—after correction) for trochanter, ilium, mid‐vertebrae (L2‐L4), and edge‐vertebrae (T11, T12, L1, L5, and S1) for 22 subjects with myelofibrosis grade > 0. Subject‐specific values are depicted by dots along the middle vertical line. Box boundaries correspond to the 1st and 3rd data quartiles (Q1 and Q3) and mid‐lines show median values. Pluses indicate outliers beyond 99% confidence intervals (error‐bars). Black horizontal lines connect the bone sites with significant differences (*p < 0.05, and Bonferroni corrected **p < 0.008) before (red asterisks) and after GNC (green asterisks). (B) Box‐plot of percent mean ADC GNC change (100% (ADC − ADC_GNC_)/ADC_GNC_) across bone sites for 22 subjects (in Figure 4A). Solid horizontal line marks zero bias.

The bias correction impact on mean ADC values of individual bone‐site combinations for 22 subjects is summarized in Table 1 and Figure 4. The GNC effect on ADC was significant (*p* < 0.001) for all individual bone sites except mid‐vertebrae (*p* > 0.12), least affected by GNL bias (Figure 2). Although ROI sizes were three‐to‐four‐times larger for trochanter and ilium than for vertebrae bodies (Table 1), no significant correlation to bone volume was detected (*p* > 0.15) for ADC and SD (ADC), indicating that mean ADC measurement were independent of ROI volumes. The ADC correlation to PDFF values in trochanter and ilium was similarly not significant (*p* > 0.15) confirming minor effect of residual unsuppressed fat on measured mean ADC values (for subjects with MF grade > 0). However, significant positive correlation (*p* = 0.02; *R* = 0.5) was detected for PDFF and SD(ADC) that likely reflected increasing ADC measurement errors for higher fat content due to limited bone marrow water SNR and CNR, eventually leading to ADC histogram truncation (Figure S2). Relative to clinical readouts (Table S2), no significant correlation to age was observed (*p* > 0.09) for ADC of all bone sites, while trochanter ADC was significantly higher for female subjects (*p* = 0.02) both before and after GNC. Correction also minimally enhanced significant correlation of ilium ADC to MF grade (*R* = 0.48, *p* = 0.03) versus before GNC (R = 0.46, *p* = 0.035).

**TABLE 1:** ADC GNC (mean ± SD) summary by bone‐site across study subjects.

Bone site	Trochanter	Ilium	Mid‐vertebrae	Edge‐vertebrae
Volume ± 0.1 (cm^3^)	88.3 ± 25.9	72.4 ± 12.5	21.7 ± 7.4	20.1 ± 6.7
ADC ± 0.001 (μm^2^/ms)				
Before GNC	0.542 ± 0.136	0.646 ± 0.203	0.580 ± 0.202	0.568 ± 0.182
After GNC	0.519 ± 0.124	0.628 ± 0.193	0.577 ± 0.199	0.591 ± 0.178
P (ADC, ADC_GNC_)	< 0.001	< 0.001	0.13	< 0.001

For majority of subjects, GNC helps confidently detect ADC heterogeneity across the bone sites (Figure 4A), manifesting in tendency for higher ilium and edge‐vertebrae ADC compared to trochanter and mid‐vertebrae. The notable improvement of ADC differentiation for trochanter versus edge‐vertebrae and ilium after GNC confirms proper correction implementation (by lowering ADC of trochanter and increasing ADC of edge‐vertebrae, Table 1). The median percent ADC nonuniformity bias corrected between edge‐vertebrae and trochanter across all subjects is 8.5%, and ranging up to 17% (Figure 4B). The nominal significance of difference between ilium and edge vertebrae ADC before GNC (*p* < 0.003) disappears after correction (*p* = 0.073) (Figure S4B), suggesting that this distinction was largely artefactual due to GNL nonuniformity bias. Interestingly, GNC emphasizes the contrast between trochanter and ilium ADC (lower *p* value, Figures 4A and S4B green) and reveals significant differences between trochanter and edge‐vertebrae (*p* = 0.0068 versus *p* = 0.17 before GNC), thus aiding accurate detection of ADC heterogeneity between individual bone sites of MF patients.

ADC correlation among the bone sites (*R* = 0.6–0.72) was significant (*p* < 0.0035) both before and after GNC, reflecting relatively consistent ADC heterogeneity trends across bone sites for majority of MF subjects (Table 1: mean ADC_GNC_ lower for femur, higher for ilium, lower for mid‐vertebrae, and higher for edge‐vertebrae). Compared to ADC before correction, GNC altered heterogeneity trends of seven subjects at one or two bone sites. The deviation from average trend (Figure 4A, Table 1) with ilium ADC lower than femur was observed for 6/22 subjects post GNC (4/22 before GNC), and ilium lower than L2‐L4 ADC for 5/22 subjects post GNC (3/22 before GNC). Twelve subjects had edge‐vertebrae ADC lower than mid‐vertebrae before GNC, which was reduced to eight subjects by GNC, indicating that 4 subjects likely had GNL‐induced ADC non‐uniformity. Interestingly, out of the eight subjects deviating from average trend, 6 had MF grade 3, suggesting that ADC heterogeneity trends (clarified by GNC) could be informative of MF disease severity.

## 4 Discussion

Our study confirmed that on‐scanner GNC provides a viable option for correction of GNL bias in ADC in a clinical trial setting. We demonstrated that methodologic improvements provided by this correction significantly increase ADC accuracy across multiple bone sites. These systematic bias corrections were essential to disentangle biologic ADC heterogeneity across pelvic bones and vertebrae of myelofibrosis patients from systematic nonuniformity errors (ranging 8.9% to 16.5%). GNC also eliminated substantial errors in ADC due to variable positioning (average bias range of ±4.2% and up to ±8.5% at the same bone site) for more robust longitudinal MF ADC measurement during therapy response monitoring. The largest errors in BM ADC were corrected for bone sites offset farthest from magnet isocenter including edge vertebrae (e.g., T12, L1, L5, S1, down to −10%) and femoral trochanter (up to +8%) due to both patient size and variable positioning. The L3 mid‐vertebrae ADC has shown minimal GNL errors and would provide the most robust BM site for bias‐free ADC measurement on scanners that lack GNC capabilities. In our study of MF patients, the GNL induced‐errors resulted in spatial nonuniformity artifactually increasing trochanter ADC and reducing edge‐vertebrae ADC. By removing these biases, GNC helped unravel biological heterogeneity trends in BM ADC across bone sites potentially related to patient gender and severity of MF disease. We also found that bone marrow ADC measurements were most reliable for higher fibrosis grade (accompanied by low PDFF) and confirmed that corrected ilium ADC values were significantly correlated to MF grade [9].

Healthy bone marrow in older adults has a substantial fat contribution and relatively low ADC [16, 17, 18]. All BM ADC studies employ fat suppression, however, imperfect quenching of the spectral component under‐lying water peak by spectral selective suppression methods may artefactually lower measured bone marrow ADC depending on the fat content [33]. Previous MRI PDFF studies reported fat‐fraction heterogeneity across bone sites in healthy bone‐marrow with fat‐fraction increasing for femur versus ilium versus vertebrae and with patient age [34, 35]. Our recent study in MF subjects [9] likewise confirmed these trends in MF BM, although with lower fat‐fractions compared to healthy BM and non‐MF myeloproliferative neoplasm. We also observed higher PDFF in a subset of subjects with low MF grade in our study and noticed that with higher fat content, BM ADC measurement became unreliable due to poor SNR and CNR of low ADC (< 0.4μm^2^/ms) measurements. This interpretation was supported by detection of significant SD(ADC) correlation to PDFF. These results also suggest limited SNR and CNR as a likely source of relatively low repeatability reported for ADC in healthy bone marrow [16, 17, 18]. Higher fat fraction in femurs may lower the observed ADC values compared to the other bone sites. However, we did not observe significant correlation between trochanter PDFF and ADC in our study for subjects with MF grade > 0. The opposite trend (positive correlation between PDFF and ADC) has also been previously reported for ilium and vertebrae of subjects with non‐metastatic tumors [11], suggesting that actual effect of BM fat content on ADC may be dependent on bone region and disease.

The question of inherent biologic ADC heterogeneity across bone marrow sites is less investigated in literature. Previous MRI studies on healthy volunteers were usually performed for a younger population with lower BM fat fraction [16, 17, 18, 36] than typical for an older MF disease population. Prior ADC studies for healthy subjects in vertebrae [33, 36] and in multiple bone sites [16, 17, 18, 37] report varying average ADC values (0.25–0.45 μm^2^/ms) largely dependent on employed fat‐suppression methods and subject age [16, 37] and typically do not consider effects of SNR and CNR detection limits or ADC histogram truncation. These studies also used varying gradient systems that did not include GNL correction further confounding derivation of accurate reference ADC values. However, except for multi‐scanner repeatability study that reported uniform ADC across femur, ilium and vertebrae [18], there is an apparent trend for marginally higher ADC values in vertebrae versus femur and similar ADC in ilium and femur of healthy subjects in literature [16, 17, 36, 37]. In two age‐matched healthy volunteers in our study, we measured ADC values of 0.3–0.4 μm^2^/ms nominally independent of bone site suggesting relative ADC homogeneity in absence of MF disease [9]. The unambiguous analysis of BM ADC homogeneity in healthy subjects requires both enhanced CNR and GNC application.

For subjects with MF grade > 0, we consistently observed ADC (0.52–0.63 μm^2^/ms) higher than in healthy bone marrow [9, 16, 17, 18, 37]. Broad range of ADC values (0.5–2.5 μm^2^/ms) substantially higher than in healthy BM was also reported by the recent studies in diseased subjects with BM metastasis from breast and prostate cancer [21, 37, 38] and focal and diffuse myeloma lesions [37, 39]. These reported values were combined across pelvis and vertebrae preventing ADC heterogeneity analysis between bone sites. Only one study applied GNC for ADC of pelvic and vertebrae BM lesions [21] that removed GNL nonuniformity errors up to 20%, comparable to our results. The investigation of BM ADC for subjects with monoclonal plasma cell disorder [40] described lower ADC values for S‐vertebrae versus ilium. This finding is consistent with our MF study observation of lower ADC for edge vertebrae before GNC that became closer to ilium ADC after GNC for majority of subjects, suggesting the uncorrected GNL bias as a possible source of nonuniform ADC in Reference [40]. We also observed higher ADC in ilium compared to L2‐L4 of MF patients post GNC. This contrasts with a previous BM DWI study of subjects with non‐metastatic tumors [11] that reported lower ADC values for ilium versus L3. Ascertaining the origin of the ADC heterogeneity trend in relation to specific disease requires elimination of spatial nonuniformity bias.

The main limitation of our study is a single‐center single‐scanner trial with a small number of participants for a relatively rare cancer (myelofibrosis). However, the demonstrated GNC workflow validation and integration for clinical imaging trial is generally applicable to bone‐marrow ADC studies. The systematic GNL nonuniformity bias in ADC is common across clinical scanners for off‐center anatomies [21, 22, 23] and is known to increase ADC errors for multi‐system studies due to added variability of gradient system characteristics [25, 26]. The integration of vendor‐provided GNC implementations into clinical trial workflow provides the most practical implementation compared to more onerous retrospective corrections [26] that rely on access to proprietary information on gradient system design and DICOM scaling [26, 29]. Our preliminary findings of nonuniform ADC for femur versus ilium and edge vertebrae in majority of MF subjects, as well as deviation of heterogeneity trends in edge vertebrae of high‐grade MF would require confirmation with a larger population study. However, our observation of significant GNL bias effect on ADC values across bone sites indicates the importance of GNC for any future investigations that aim to assess inherent ADC heterogeneity in bone marrow induced by disease. Correction of GNL errors due to varying patient positioning would also be beneficial for the longitudinal studies that use ADC to monitor therapy response for individual patients with heterogeneous disease.

## 5 Conclusion

The study demonstrated the value of on‐scanner ADC correction for GNL bias in an imaging trial. While vendor‐provided GNC implementation for spatial b‐value bias is not yet widely adopted, it is analogous to automatic geometric distortion correction which is standard in routine MRI and clinical trials that utilize ADC. This work also revealed the importance of GNC for determination of biologic versus artifactual heterogeneity in ADC across bone‐marrow sites. Notable correction impact on BM ADC values promises improvements of accuracy, uniformity and longitudinal reproducibility for diagnostic and prognostic thresholds sought by trials that employ quantitative diffusion imaging. The improvements achieved will help design future studies to formulate and test specific hypothesis for relations between bone marrow ADC and clinical readouts.

## Funding

This work was supported by National Institutes of Health (R01CA190299, R01CA238023, R01CA297995, U24CA237683).

## Conflicts of Interest

Dariya Malyarenko is a co‐author of IP (US9851426B2) that underlays described GNC method assigned to and managed by the University of Michigan and licensed by Philips MR and Siemens Healthineers. Thomas L. Chenevert is a co‐author of IP (US9851426B2) that underlays described GNC method assigned to and managed by the University of Michigan and licensed by Philips MR and Siemens Healthineers. Brian D. Ross is a co‐author of IP (US9851426B2) that underlays described GNC method assigned to and managed by the University of Michigan and licensed by Philips MR and Siemens Healthineers. Johannes M. Peeters is an employee of Philips Healthcare. Ajit Devaraj is an employee of Philips Clinical Science. Ramin Jafari is an employee of Philips Clinical Science.

## Supporting information


**FIGURE S1:** Illustration of on‐scanner ADC_GNC_ (A) validation for the uniform sodium polyacrylate gel (SPAG) phantom in an oblong acrylic shell (B). The room temperature ADC_SPAG_ = 2.13 μm^2^/ms at isocenter is restored by GNC within coronally imaged FOV (400 × 200 mm^2^, white outline) with S‐I axis running horizontally. (C) Demonstration of good agreement (< 1% voxel‐wise differences) between measured fractional GNL bias (top: %$\left(\mathrm{ADC}-{\text{ADC}}_{GNC}\right)/{\text{ADC}}_{GNC}$) and system spherical harmonics (SPH) model (bottom: %(C_b_‐1)). Color‐bars display the value scales for the corresponding maps. (D) Phantom ADC histograms (normalized to total volume) are compared before and after GNC. ADC GNC formalism summarized under histograms for b‐maps, $b\left(\vec{r}\right)$, GNL tensor, $\hat{L}$, and orthogonal DWI directions, $u_{k}$.
**Figure S2:** Ilium ADC histograms (bin size 0.05μm^2^/ms, normalized to total counts) for subjects with ilium fat fractions FF = 20%, 30%, 38% and 53% (color‐coded in the legend)) illustrate apparent detection truncation for ADC < 0.1 μm^2^/ms (FF > 35%). The histogram mode is 0.22 μm^2^/ms (< median (0.31 μm^2^/ms) < mean (0.35 μm^2^/ms)) both for MF subject with FF = 38% and healthy control with FF = 53%. The ADC histograms for trochanter of healthy subjects (not shown, mean ADC = 0.35 ± 0.23 mm^2^/ms) also overlap with their ilium ADC histograms. Confident ADC measurement is evidently possible for FF < 35% with ADC histogram median ˜ mean ˜ mode.
**Figure S3:** Bland–Altman plots for GNL bias (33 test–retest pairs) for combined bone sites of left and right trochanter (A), left and right ilium (B), L2 and L3 vertebrae (C), L1 and L5 vertebrae (D) (circles color‐coded in the legends) The dashed lines correspond to 95% limits of agreements across combined bone sites with respect to average bias marked by a solid line.
**Figure S4:** (A) Mean ADC and ADC_GNC_ trends (color‐coded in the legend) across trochanter, ilium, mid‐vertebrae (Mid‐Vert), and edge‐vertebrae (Edge‐Vert) bone marrow sites for an example subject (in Figure 3). Dashed lines are added for visual guidance to underscore higher uniformity of ADC across bone sites after correction. Inserts show axial ADC maps with common color‐bar before and after GNC for ROIs of right trochanter (top, positive bias) and T12 (bottom, negative bias). (B) Log‐P values for single‐sided t‐test of ADC between bone sites (T: trochanter; I: ilium; EV: edge‐vertebrae (T11, T12, L1, L5, S1) and MV: mid‐vertebrae (L2‐L4)) before and after GNC (color‐coded in the legend). The dashed lines mark the *p* = 0.05 (black) and Bonferroni‐corrected *p* = 0.0083 (cyan) significance thresholds, respectively.
**Table S1:** Scan parameters
**Table S2:** Study demographics.

## Data Availability

The data that support the findings of this study are available from the corresponding author upon reasonable request.
