# Supplementary material for: On‐Scanner Correction of Gradient Nonlinearity Bias for Accurate Assessment of Diffusion Heterogeneity Across Bone Sites in Myelofibrosis Patients
Source: Magn Reson Med. 2026 Jan 29;95(6):3386–95. doi: 10.1002/mrm.70273 (PMC13049273; doi:10.1002/mrm.70273)

**Supplementary Materials**

*ROI definition:*

Bone marrow regions-of-interest (ROIs) (e.g., Figure 1) for right ilium (RI), left ilium (LI), right trochanter (RT), and left trochanter (LT) were manually delineated on in-phase (IP) PDFF images of lower (pelvic) station for the 1^st^ imaging point of all subjects using 3D Slicer as described (28). The delineations were limited to the thick parts of the posterior ilium and femoral trochanter bones (Fig.1, insert) to ensure sufficient BM area (excluding narrow iliac wings) and consistent coverage (excluding femoral shafts) by DWI. For five subjects having longitudinal scans, baseline ROI masks were propagated to later time points as follows: (1) coordinates of fiducial points were defined and recorded in specific order on 6 landmarks of a slice through the iliac crest and 8 landmarks on a central slice through the femoral heads for each timepoint; (2) Matlab (R2019b) function “*procrustes*” was used to determine the spatial linear transformation that mapped baseline fiducials onto subsequent timepoint fiducials; (3) the same transformation was then applied to the baseline ROI mask to create ROI masks at each timepoint. Additional ROIs were defined semi-automatically for lumbar and thoracic vertebral bodies on the superior DWI station using manually defined fiducials at the center of each vertebral body on ADC maps. A custom Matlab (R2019b) script was used to convert minimal right-left and anterior-posterior size of vertebrae combined with their fiducial centers into elliptical cylindrical masks conservatively well within vertebral bodies. All masks were saved as “label” files in MHD format. All semi-automated annotations were visually inspected, and fiducial definitions were performed in 3D Slicer ver 4.6.2.

Supporting Table S1. Scan parameters

| Sequence parameters | DWI | PDFF |
| --- | --- | --- |
| Fat-suppression | STIR (gradient reversal; TI=220ms) | mDIXON-QUANT |
| FOV (mm^2^) | 450 | 420 |
| TR (ms) | 7200 | 6.1 |
| TE (ms) | 81 | 1.04, 1.84, 2.64, 3.44, 4.24, 5.04 |
| Flip angle (^o^) | 90 | 3 |
| Resolution (mm^3^) | 3x3x5 | 2x2x3.5 |
| b-values (NEX) (s/mm^2^) | 0 (5)  800 (10) | NA (1) |
| Time (min) | 4.5-5 | 0.25 |

Supporting Table S2. Study demographics

| analysis type | No. Subjects | Age (yrs)  Median [range] | No. Male / Female | No. G3 / G2 / G1 / G0 / HC / NA |
| --- | --- | --- | --- | --- |
| b-map | *41 | 63 [29,82] | 15 / 26 | 7 / 8 / 12 /  6 / 2 / 6 |
| ADC | **22 | 64 [29,79] | 8 / 14 | 7 / 7 / 6 /  1 / 0 / 1 |

No: number; G0-G3: MF grade from iliac biopsy; HC: healthy controls; NA: not available.

*Twenty out of the forty-one study subjects were previously analyzed in (9) though without regard to GNL effects. ** Nine out of the twenty-two subjects overlap with the study in (9).


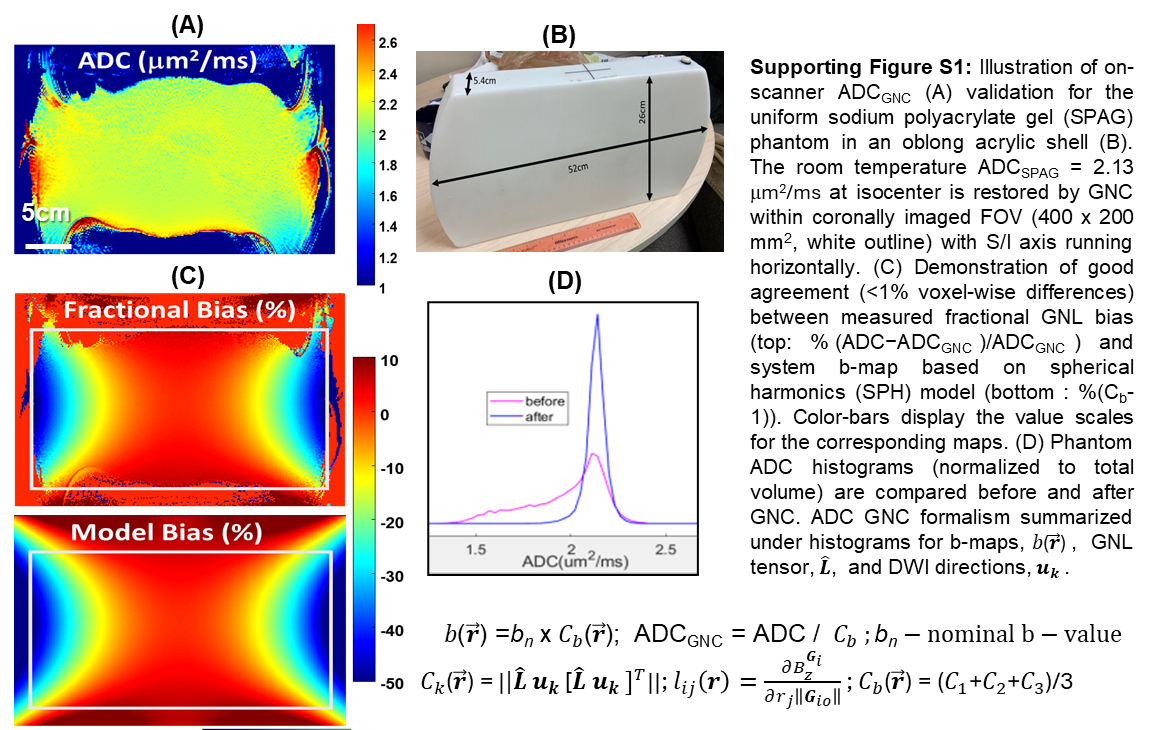


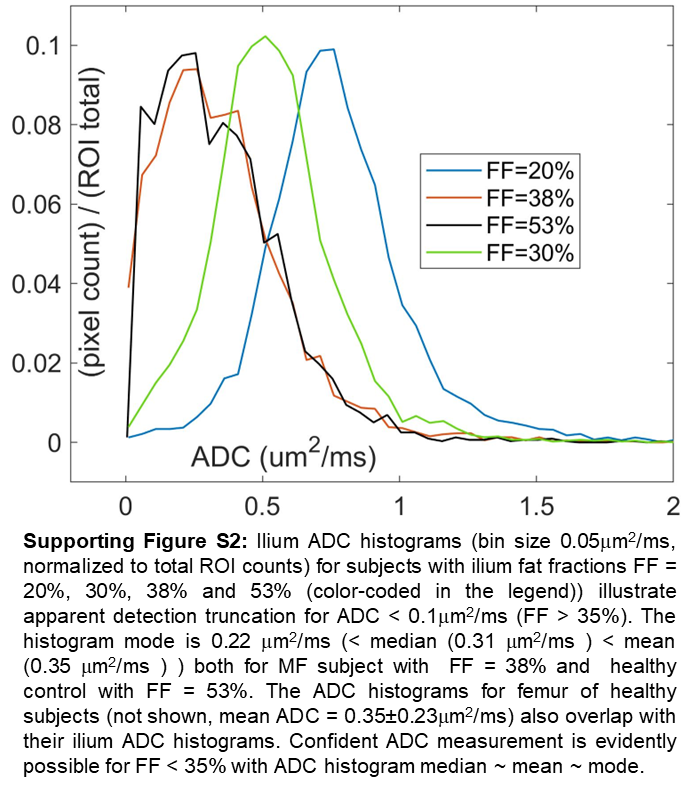


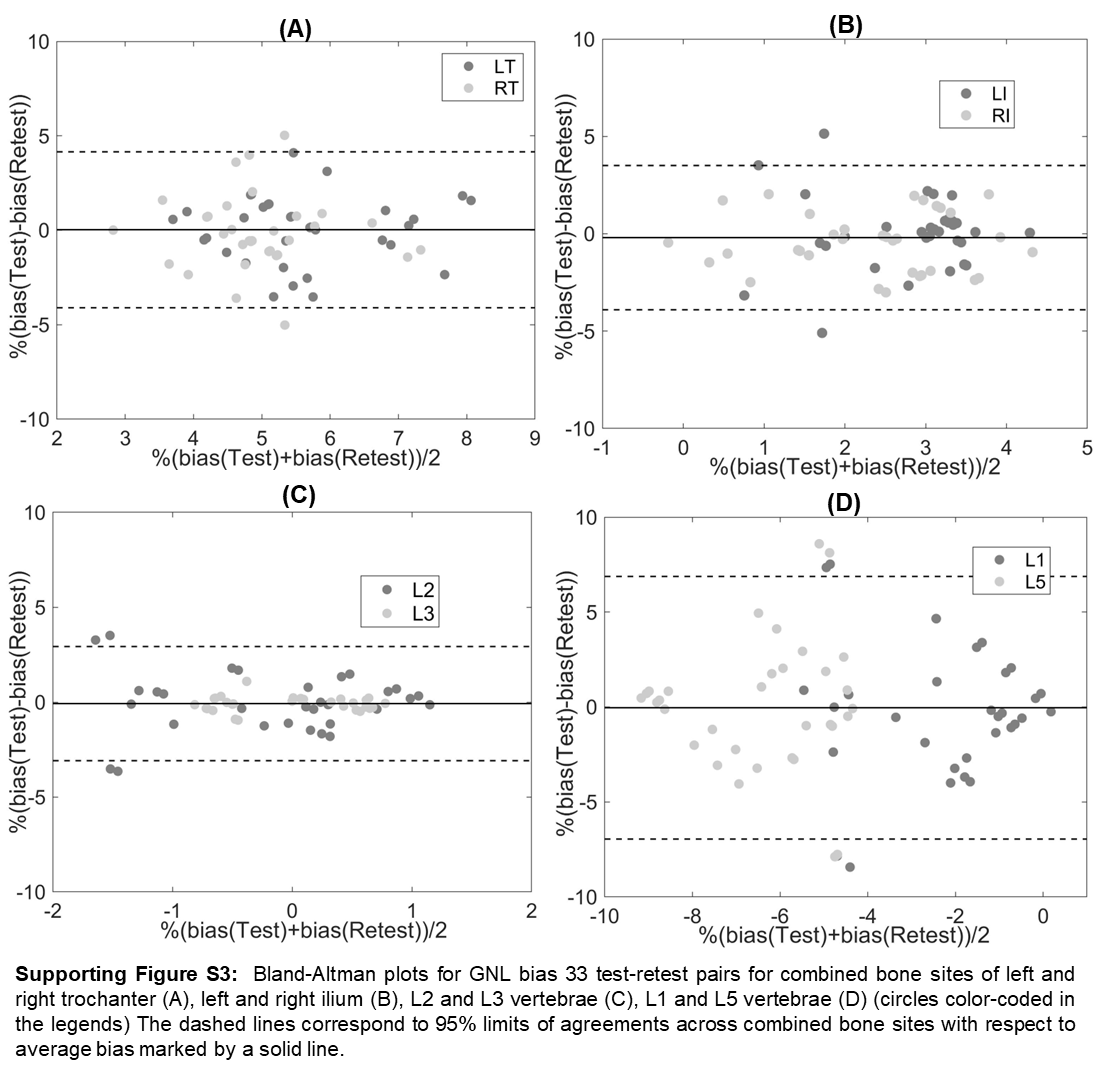


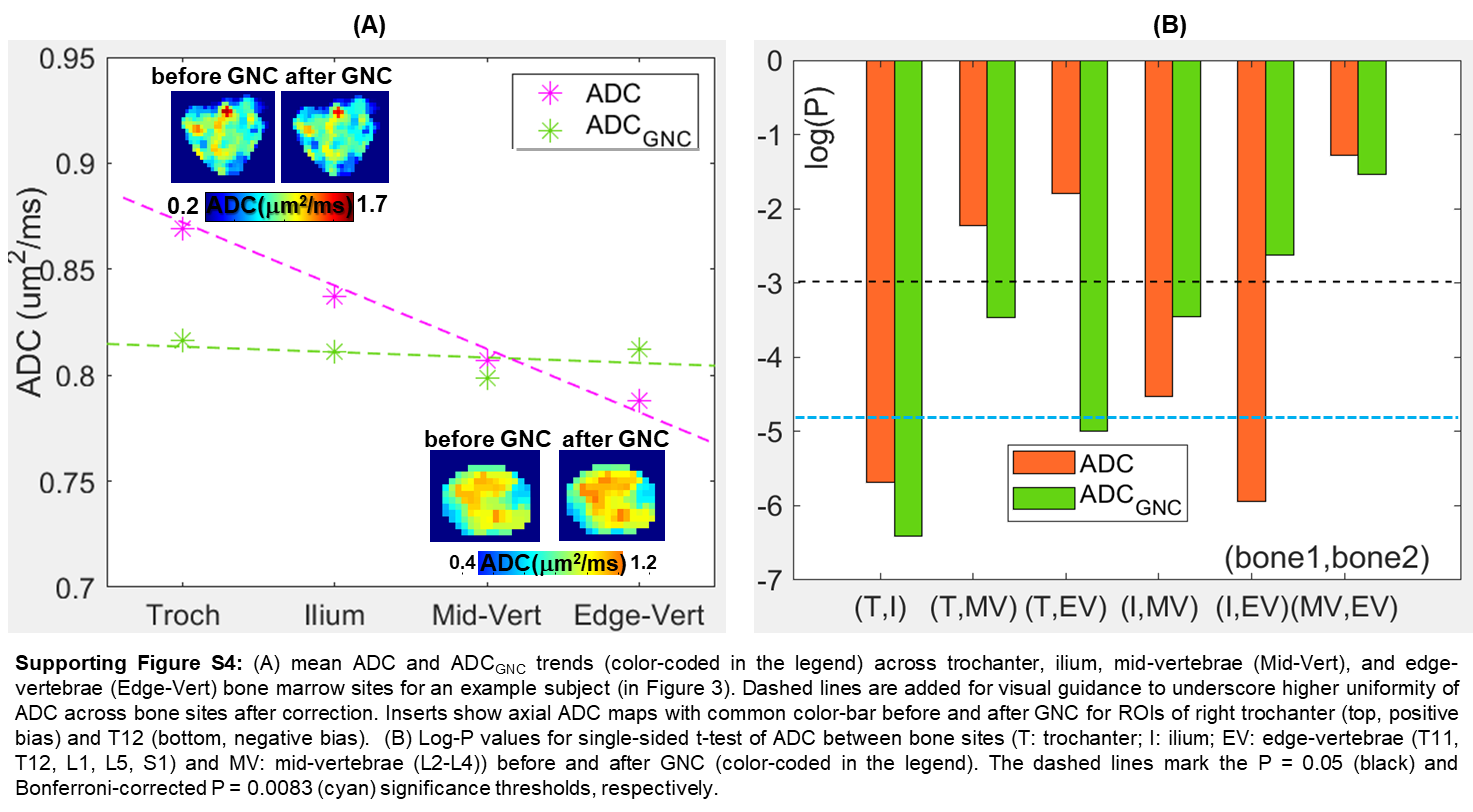

Supplement: Supplementary file 1 — FIGURE S1: Illustration of on‐scanner ADCGNC (A) validation for the uniform sodium polyacrylate gel (SPAG) phantom in an oblong acrylic shell (B). The room temperature ADCSPAG = 2.13 μm2/ms at isocenter is restored by GNC within coronally imaged FOV (400 × 200 mm2, white outline) with S‐I axis running horizontally. (C) Demonstration of good agreement (< 1% voxel‐wise differences) between measured fractional GNL bias (top: %ADC−ADCGNC/ADCGNC) and system spherical harmonics (SPH) model (bottom: %(Cb‐1)). Color‐bars display the value scales for the corresponding maps. (D) Phantom ADC histograms (normalized to total volume) are compared before and after GNC. ADC GNC formalism summarized under histograms for b‐maps, b(r→), GNL tensor, L^, and orthogonal DWI directions, uk. Figure S2: Ilium ADC histograms (bin size 0.05μm2/ms, normalized to total counts) for subjects with ilium fat fractions FF = 20%, 30%, 38% and 53% (color‐coded in the legend)) illustrate apparent detection truncation for ADC < 0.1 μm2/ms (FF > 35%). The histogram mode is 0.22 μm2/ms (< median (0.31 μm2/ms) < mean (0.35 μm2/ms)) both for MF subject with FF = 38% and healthy control with FF = 53%. The ADC histograms for trochanter of healthy subjects (not shown, mean ADC = 0.35 ± 0.23 mm2/ms) also overlap with their ilium ADC histograms. Confident ADC measurement is evidently possible for FF < 35% with ADC histogram median ˜ mean ˜ mode. Figure S3: Bland–Altman plots for GNL bias (33 test–retest pairs) for combined bone sites of left and right trochanter (A), left and right ilium (B), L2 and L3 vertebrae (C), L1 and L5 vertebrae (D) (circles color‐coded in the legends) The dashed lines correspond to 95% limits of agreements across combined bone sites with respect to average bias marked by a solid line. Figure S4: (A) Mean ADC and ADCGNC trends (color‐coded in the legend) across trochanter, ilium, mid‐vertebrae (Mid‐Vert), and edge‐vertebrae (Edge‐Vert) bone marrow sites for an example subject (in Figure [file MRM-95-3386-s001.docx]
